# Adverse Effect of Antifouling Compounds on the Reproductive Mechanisms of the Ascidian *Ciona intestinalis*

**DOI:** 10.3390/md11093554

**Published:** 2013-09-20

**Authors:** Alessandra Gallo, Elisabetta Tosti

**Affiliations:** Laboratory of Animal Physiology and Evolution, Stazione Zoologica Anton Dohrn, Villa Comunale, Naples 80121, Italy; E-Mail: alessandra.gallo@szn.it

**Keywords:** TBT, diuron, antifouling compounds, ascidians, gametes, fertilization, larval development, ion currents

## Abstract

Fertilization and embryo development that occur in sea water are sensitive to xenobiotics from anthropogenic sources. In this work, we evaluated the influence of two antifouling biocides, tributyltin (TBT) and diuron, on the reproductive mechanisms of the marine invertebrate *Ciona intestinalis*. By using electrophysiological techniques, we examined the impact of these compounds on the electrical properties of the mature oocytes and of events occurring at fertilization. With different toxicity assays, we studied the effect of the two biocides on the gametes by evaluating fertilization rate and embryo development. Results show that sodium (Na^+^) currents were significantly reduced by either of the two biocides, whereas conductance was significantly increased. The fertilization current frequency and amplitude, fertilization rate and larval development were affected only by TBT. This study suggests that: (i) the two biocides affect either the electrical properties of the oocyte plasma membrane and the reproductive success representing a risk factor for the survival of the species exposed to environmental pollution; (ii) the ascidian *Ciona intestinalis* may represent a good model organism to test toxicity of marine pollutants. Possible mechanisms of action of the two biocides are discussed.

## 1. Introduction

Environmental pollution represents a serious hazard for terrestrial and aquatic ecosystems and may result in significant ecotoxicological effects. Over the last decades, diverse substances have been released into marine environment as a consequence of agricultural production, manufacturing processes and their by-products. These chemicals include herbicides, pesticides, fungicides, plasticizers, antifoulants and others [1]. There is increasing evidence that a number of chemicals in the environment may affect reproductive function and disrupt the endocrine systems of aquatic life and wildlife [2,3,4,5,6,7]. Among the chemicals able to disrupt endocrine functions in marine invertebrates, organotin compounds in gastropods play a relevant role. Several studies report the diffusion of female marine snails with male genitalia, including a penis and vas deferens [8,9,10]. This phenomenon, known as imposex, appears to be an irreversible induction of male sex characteristics on females and was proved to be caused mainly by exposure to tributyltin (TBT) compounds [11,12]. TBT is a biocide widely used in antifouling paints and in agricultural and industrial applications, this leads to its direct marine environmental input [13]. Among the acute negative effects exerted by TBT are included DNA damages, apoptosis induction, genotoxicity, neurotoxicity, inhibition of post fertilization cleavage and embryo development arrest [14]. Furthermore, a specific toxic effect of TBT has been reported on cell membranes of mitochondria and erythrocytes [14,15].

Following this crescent alarm on the TBT toxicity, the International Maritime Organization [16] adopted the convention for the control of dangerous antifouling system on ships (AFS) that prohibited the application of TBT-based antifouling paints by 1 January 2003 and required the absence of such paints on the surface of vessels by 1 January 2008. However, nonetheless this international prohibition, TBT is still regularly used in countries that have not adopted AFS convention [17]. For this reason, there is currently considerable interest in understanding the mechanisms of action of TBT.

As an alternative to organotin compounds, organic booster biocides were introduced in antifouling paint formulation, among which diuron (3-(3,4-dichlorophenyl)-1,1-dimethylurea) is the most widely employed [18]. Consequently, the persistence of diuron and TBT in marine ecosystem is due to their high stability in water column and accumulation in the sediments [19,20]. Their presence may impact aquatic organisms and in particular the invertebrates, that is alarming in view of the ecological importance of invertebrates that are key components of all ecosystems [21]. In ascidians, the impact of organotins on the reproductive processes has been widely studied [22,23,24,25]. In the ascidian *Ciona intestinalis*, the physiology of reproduction is well known and in particular, the electrical characteristics of the oocyte and embryo plasma membrane and the ions involved in fertilization have been well described [26,27,28]. The mature oocyte is blocked at metaphase I (MI), characterized by the presence of sodium (Na^+^) currents and ready to be fertilized. The first electrical modification of the oocyte at fertilization is the generation of a fertilization current (FC) due to the gating of nonspecific ion channels [29,30] that may be modulated by different stimuli [31]. The first mitotic division of the zygote occurs after 50 min from fertilization and from the two cell stage divisions occur every 35 min up to a swimming larva that hatches from the extracellular investments after 24 h. Larval stage is a transient period that lasts 48 h, after that the metamorphosis into a sessile adult organism starts.

In this study, we have evaluated and compared the effects of TBT and diuron, as old and new generation antifouling biocides, on the oocyte physiology, fertilization and embryo development of *C. intestinalis*, by using: (i) electrophysiological techniques to record ion currents and electrical events at the level of oocyte plasma membrane; (ii) different biological toxicity assays on gametes and embryos.

## 2. Results

### 2.1. Effects of TBT and Diuron on the Plasma Membrane Conductance

**Figure 1:**
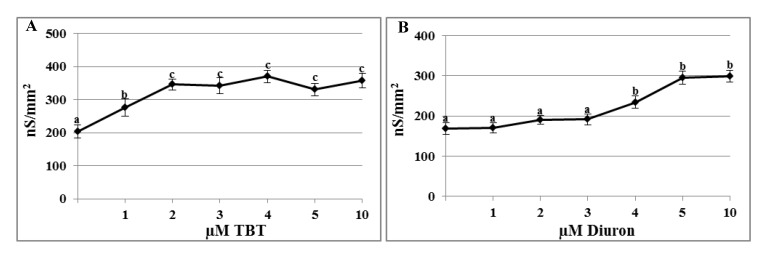
Effects of tributyltin (TBT) and diuron on plasma membrane conductance. Conductance recorded in oocytes incubated for 30 min with different concentrations of TBT (**A**) and diuron (**B**) significantly increased. Data were normalized per mm^2^ and reportedas mean ± SE. ^a^^,b^^,c^ Different superscripts denote highly significant difference, *p* < 0.01.

The plasma membrane conductance recorded in untreated oocyte was 203.04 ± 20.40 nS/mm^2^ and significantly increased after incubation in 1 µM TBT (276.09 ± 26.41 nS/mm^2^; *p* < 0.01). At concentration of 2 µM, conductance still increased and, from this value, it reached a plateau (345.46 ± 17.08 nS/mm^2^ at 2 µM; 341.80 ± 23.87 nS/mm^2^ at 3 µM; 369.49 ± 18.32 nS/mm^2^ at 4 µM; 330.21 ± 18.97 nS/mm^2^ at 5 µM; 357.22 ± 21.40 nS/mm^2^ at 10 µM; *p* < 0.01) (Figure 1A).

The conductance recorded in oocytes incubated in 1–3 µM diuron were not different from the control (control 168.42 ± 14.2 nS/mm^2^; 170.2 ± 12.4 nS/mm^2^ at 1 µM; 190.5 ± 11.2 nS/mm^2^ at 2 µM and 191.3 ± 13.1 nS/mm^2^ at 3 µM), whereas at 4 µM it significantly increased (233.7 ± 15.3 nS/mm^2^; *p* < 0.01). When oocytes were incubated in 5 µM diuron, conductance increased again and reached a plateau at the highest test concentrations (295.5 ± 16.2 nS/mm^2^ at 5 µM and 298.7 ± 14.8 nS/mm^2^ at 10 µM; *p* < 0.01) (Figure 1B).

### 2.2. Effects of TBT and Diuron on Na^+^ Currents

**Figure 2:**
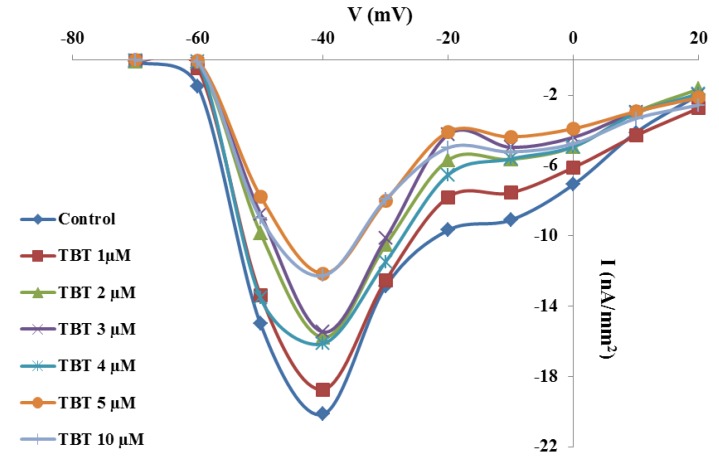
Effects of TBT on Na^+^ current amplitude. Mean I/V curves constructed from current peak values recorded in oocytes clamped from the holding potential of −80 mV to the test potentials between −70 mV and +20 mV after incubation for 30 min with different TBT concentrations. Error bars indicating the SE were omitted for image clarity.

I/V curves constructed from oocytes clamped at different voltage values and treated with the test concentrations of TBT showed that this biocide reduced, in concentration-dependent manner, the amplitude of Na^+^ current starting from the concentration of 2 µM (Figure 2). The maximum peak of Na^+^ currents in untreated oocytes was 20.14 ± 0.3 nA/mm^2^ and did not change significantly after incubation in 1 µM TBT (18.73 ± 0.5 nA/mm^2^). After incubation in 2–4 µM TBT, Na^+^ current amplitude decreased significantly in comparison to the control (*p* < 0.01), even if there was no significant difference between these test concentrations (15.76 ± 0.2 nA/mm^2^ at 2 µM; 15.46 ± 0.4 nA/mm^2^ at 3 µM; 16.13 ± 0.4 nA/mm^2^ at 4 µM). At concentration of 5 µM the amplitude of Na^+^ currents decreased again, and, from this value, it reached a plateau (12.14 ± 0.6 nA/mm^2^ at 5 µM and 12.26 ± 0.5 nA/mm^2^ at 10 µM; *p* < 0.01).

**Figure 3:**
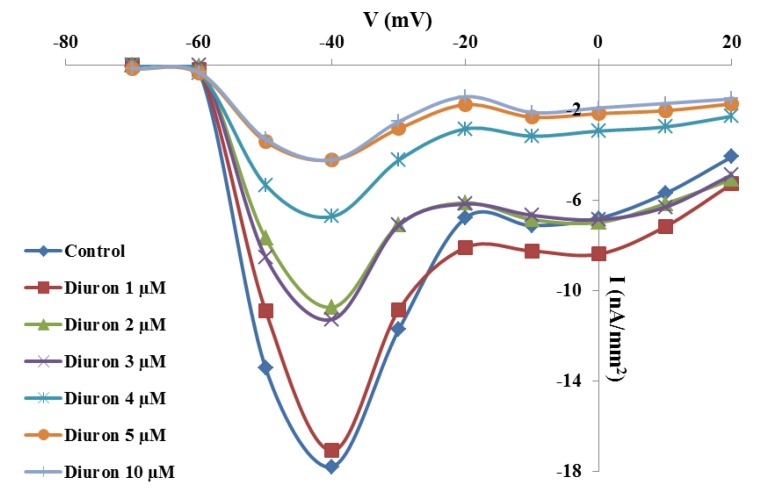
Effects of diuron on Na^+^ current amplitude. Mean I/V curves constructed from current peak values recorded in oocytes clamped from the holding potential of −80 mV to the test potentials between −70 mV and +20 mV after incubation for 30 min in different diuron concentrations. Error bars indicating the SE were omitted for image clarity.

In addition, diuron reduced the amplitude of Na^+^ currents in a concentration-dependent manner starting from concentration of 2 µM (Figure 3). Na^+^ current amplitude recorded in untreated oocytes (17.80 ± 0.2 nA/mm^2^) was not significantly different from that recorded after incubation in 1 µM diuron (17.09 ± 0.6 nA/mm^2^). At 2 and 3 µM we observed that the amplitude of Na^+^ currents decreased significantly in comparison to the control, with no significant differences between these two test concentrations (respectively 10.74 ± 0.4 nA/mm^2^ and 11.28 ± 0.2 nA/mm^2^; *p* < 0.01). After incubation in 4 µM diuron, the amplitude was lower (6.71 ± 0.5 nA/mm^2^; *p* < 0.01) compared to untreated and oocytes treated with 2 and 3 µM. The highest diuron test concentrations (5 and 10 µM) reduced significantly again Na^+^ current amplitude (respectively 4.21 ± 0.3 nA/mm^2^ and 4.2 ± 0.5 nA/mm^2^; *p* < 0.01).

### 2.3. Effects of TBT and Diuron on Na^+^ Channel Time Constants

The time constants of Na^+^ channels were measured by fitting the current trace recording at the test potential of −40 mV with an exponential function. Activation time constants were not significantly affected by TBT or diuron incubation at all test concentrations.

In untreated oocytes, the activation time constant had an average value of 0.98 ± 0.06 ms and did not change after TBT incubation at concentration between 1–5 µM and 10 µM (respectively 1.09 ± 0.05 ms; 1.08 ± 0.05 ms; 1.15 ± 0.07 ms; 1.04 ± 0.04 ms; 1.1 ± 0.06 ms and 1.4 ± 0.04 ms).

Similarly, after incubation in diuron at the concentration between 1–5 µM and 10 µM the activation kinetics of Na^+^ channels resulted unmodified (respectively 1.2 ± 0.04 ms; 1.1 ± 0.05 ms; 1.11 ± 0.06 ms; 1.19 ± 0.07 ms; 1.15 ± 0.07 ms; 1.2 ± 0.05 ms) with respect to the control (1.12 ± 0.05 ms).

**Figure 4:**
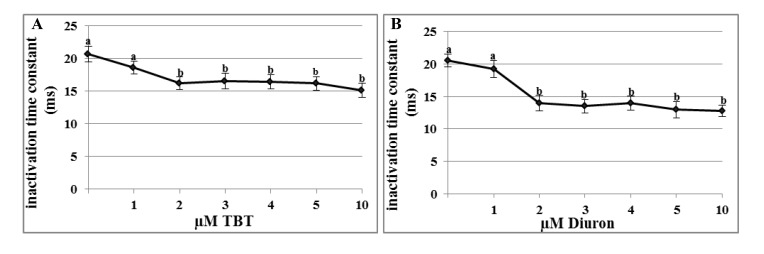
Effects of TBT and diuron Na^+^ channel time constants. Inactivation time constants were calculated at the test voltage of −40 mV after incubation for 30 min with different TBT (**A**) and diuron (**B**) concentrations. Data were reported as mean ± SE. ^a,b^ Different superscripts denote highly significant difference, *p* < 0.01.

On the contrary, the inactivation time constants decreased after incubation in 2 µM TBT or diuron. In untreated oocytes and incubated with 1 µM TBT, the inactivation time constants did not change (20.68 ± 1.2 ms and 18.61 ± 1.2 ms, respectively). After incubation in 2 µM TBT, Na^+^ current inactivated more rapidly (16.2 ± 0.9 ms; *p* < 0.01) and, from this value, it reached a plateau (16.52 ± 1.2 ms at 3 µM; 16.4 ± 1.1 ms at 4 µM; 16.17 ± 1 at 5 µM ms and 15.15 ± 1.1 ms at 10 µM; *p* < 0.01) (Figure 4A).

Also incubation in 1 µM diuron (19.2 ± 1.3 ms) did not affect the inactivation time constant in comparison to the control (20.53 ± 1 ms). After incubation with 2 µM diuron, the constant decreased significantly (13.98 ± 1.2 ms; *p* < 0.01) and, from this value, it reached a plateau (13.5 ± 1 ms; 13.96 ± 1.1 ms; 12.95 ± 1.3 ms and 12.8 ± 09 ms; *p* < 0.01) (Figure 4B).

### 2.4. Effects of TBT and Diuron on the Fertilization Current

**Figure 5:**
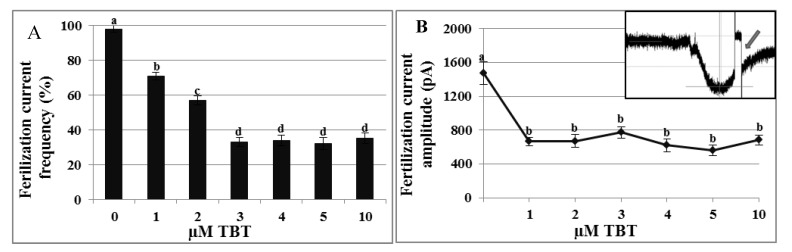
Effects of TBT on fertilization current (FC) frequency and amplitude. After TBT treatment, in oocyte clamped at −80 mV and fertilized the frequency of the FC was reduced in a concentration-dependent manner (**A**) and FC amplitude (**B**) was significantly reduced from the test concentration of 1 µM. Inset shows a control FC trace in *C. intestinalis* of ca 1400 pA amplitude; arrow indicates the interruption of recording to measure the membrane potential. ^a,b,c,d^ Different superscripts denote highly significant difference, *p* < 0.01.

In mature oocytes clamped at −80 mV, incubated with 1–3 µM TBT and then fertilized, the frequency of the FC significantly decreased (respectively 71 ± 2.3%; 57 ± 2.6%; 33 ± 2.7%) in comparison to the control (98 ± 2%). By increasing TBT concentration, the FC frequency did not change again (34 ± 3.1% at 4 µM; 32 ± 3.5% at 5 µM and 35 ± 3% at 10 µM) (Figure 5A). Mature oocytes fertilized in NSW generated a FC of 1472.80 ± 130.93 pA amplitude (Figure 5B inset). Incubation with TBT significantly reduced the amplitude of the FC at all the test concentrations (664 ± 50.4 pA at 1 µM; 669.37 ± 76.8 pA at 2 µM; 771.42 ± 69.59 pA at 3 µM; 621.62 ± 74.89 pA at 4 µM; 561.7 ± 61.14 pA at 5 µM; 682.16 ± 61.13 pA at 10 µM; *p* < 0.01) (Figure 5B).

Diuron incubation at all test concentrations did not affect significantly the frequency of FC nor its amplitude (data not shown).

### 2.5. Toxicity Assays

#### 2.5.1. Exposure of Spermatozoa to TBT and Diuron

No significant effects on fertilization rate and percentage of normal larvae were observed after fertilization of untreated oocytes with spermatozoa incubated for 30 min with TBT or diuron at concentrations between 1–5 µM and 10 µM (data not shown).

#### 2.5.2. Exposure of Oocytes to TBT and Diuron

Oocytes incubated for 30 min with TBT or diuron at concentrations between 1–5 µM and 10 µM, washed and then fertilized, did not show significant differences compared to the control for fertilization rate and normal larvae percentage (data not shown).

#### 2.5.3. Fertilization Test

Significant effects on the fertilization rate and normal larvae percentage were observed when untreated oocytes were fertilized with untreated spermatozoa in TBT solution.

**Figure 6:**
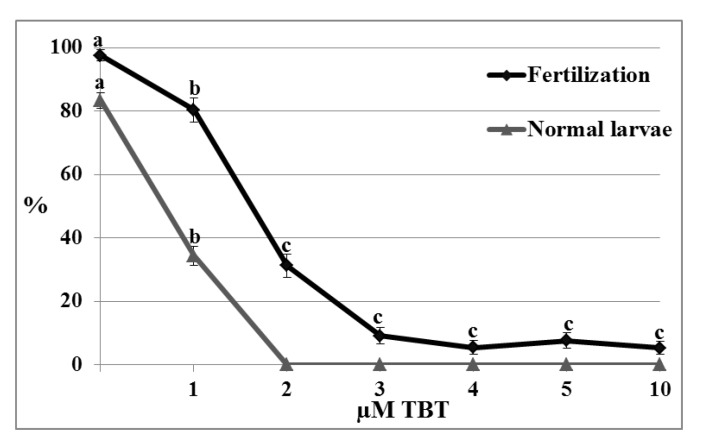
Effect of TBT on fertilization and larval development. Fertilization rate and percentage of normal larvae obtained by oocyte fertilized in natural seawater (NSW) containing TBT decreased in concentration-dependent manner. Data were reported as mean ± SE. ^a,b,c^ Different superscripts denote highly significant difference, *p* < 0.01.

**Figure 7:**
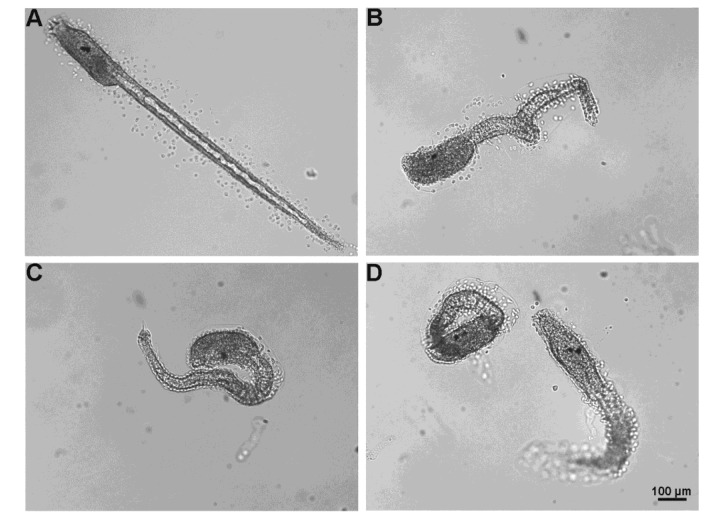
Effects of TBT and diuron on larval development. (**A**) Normal hatched larva at 24 h post fertilization developed from oocyte fertilized in NSW. Abnormal hatched larvae developed from oocyte fertilized in NSW containing TBT (**B** and **C**) or diuron (**D**) characterized by various types of tail malformations.

Fertilization rate significantly decreased in concentration-dependent manner (control 97.47 ± 1.8%; 80.35 ± 3.8% at 1 µM; 31.25 ± 3.6%; at 2 µM; 9.17 ± 2.6% at 3 µM; 5.43 ± 2.2% at 4 µM; 7.61 ± 2.5% at 5 µM and 5.27 ± 2% at 10 µM; *p* < 0.01). Embryo coming from oocytes fertilized in NSW containing 1 µM TBT developed to normal larvae in percentage significantly lower than control (83.29 ± 2.5% and 34.35 ± 3.1%; *p* < 0.01) (Figure 6). At test concentrations higher than 1 µM all the larvae resulted morphologically abnormal especially for tail malformations (Figure 7B,C).

On the contrary, diuron at concentrations between 1 and 5 µM did not affect the fertilization rate (control 98.5 ± 1%; 97 ± 1.4% at 1 µM; 98.2 ± 2.1 at 2 µM; 97.6 ± 1.9% at 3 µM; 98 ± 1.8% at 4 µM and 97.6 ± 1.9 at 5 µM) and the percentage of normal larvae (control 89.1 ± 1.4%; 86.2 ± 2.2 at 1 µM; 87 ± 1.8% at 2 µM; 86.5 ± 1.5% at 3 µM; 85 ± 1.9% at 4 µM; 85.1 ± 1.7% at 5 µM), however at the highest test concentration (10 µM), even if fertilization rate was not affected (98.2 ± 1.3%), the percentage of normal larvae significantly decreased in comparison to the control (77.7 ± 1.3%; *p* < 0.01) (data not shown). The observed abnormal larvae mainly showed tail anomalies (Figure 7D).

## 3. Discussion

Gametes of marine animals with external fertilization are exposed to water pollutants that may alter their normal physiology, which in turn may affect fertilization success. In the present study, we have shown an impact of two antifouling biocides on the physiology of the oocyte and subsequent fertilization process of the marine invertebrate *C. intestinalis*.

The role of ion currents in the oocyte physiology is well described [32,33,34,35,36]. In fact, electrical modifications of gametes are involved in maturation, activation, and fertilization due to modulation of ion channels located on the plasma membrane. In the *C. intestinalis* oocytes, the presence and modifications of voltage-dependent currents in the mature oocyte have been well described. In particular, the characterization of plasma membrane ionic asset from mature and fertilized oocytes up to the 8-cell embryo, revealed that Na^+^ currents first appear at the MI stage, remaining high up to the zygote stage. After that, Na^+^ currents decrease in the 2- and 4-cell stages up to a second increased activity at the 8-cell stage [28].

Here, we have shown that TBT and diuron reduced the amplitude of plasma membrane Na^+^ currents and the time constants for channel inactivation, whereas they increased the plasma membrane conductance of the mature oocyte. On the contrary, the biocides differently acted on electrical fertilization events, in fact only TBT exerted a negative impact on the FC by reducing either its frequency or amplitude.

In marine animals, it was reported that the permeability of the oocyte plasma membrane is altered by the interaction with TBT [37] and that an increase of membrane permeability may be due to the alteration of the molecular structure of the plasma membrane. However also diuron has been shown to perturb the plasma membrane in somatic cells [38]. These data suggest that the two biocides may share a common mechanism of action since they are inserted and accumulate into the plasma membrane phospholipids due to their lipophilic properties [15]. Our results showing the conductance increase of the plasma membrane support such previous findings due to the close relationship that exists between ion conductance and plasma membrane permeability.

Of particular interest is the significant reduction of the Na^+^ currents amplitude exerted by either the two biocides. Although this data are in agreement with Franchet *et al.* [39], who demonstrated that TBT reduced voltage-dependent Na^+^ currents up to 60% in the oocyte of the ascidian *Phallusia mammilla*, to our knowledge, a similar impact has not been previously documented for diuron. In the *C. intestinalis* oocytes, the reduction of Na^+^ currents adds an important hint on the action of TBT and diuron on the reproductive processes due to the fundamental role played by Na^+^ currents in the physiology of the mature oocyte. The alteration of Na^+^ channel function may be due to a disorganization of plasma membrane or to a direct effect on the channel [39]. The latter seems to be supported by the alteration of inactivation time constant of Na^+^ channel. In the case of TBT it has been suggested that it acts through the attraction between its positive charges and the negative ones present on the transmembrane pore entrance of the channel resulting in the occlusion of the pore [39]. On the contrary, a similar mechanism of action is not feasible for diuron as this compound does not present positive charges, possibly going to compete with the other sites of the ion channel.

In this study, TBT, but not diuron, interferes with the frequency and amplitude of FC supporting a different affinity of the two biocides with the population of specific channels that generate the FC.

In other ascidians and sea urchins, the fertilizing capacity of spermatozoa resulted in being influenced by the action of TBT and diuron [40,41]. In *C. intestinalis*, we did not observe any paternal effect, nor the pretreatment of the oocytes with the two biocides influenced fertilization and larval development. However, this is not the case for the fertilization test. In fact, the oocytes fertilized in sea water containing TBT underwent a concentration-dependent reduction of the fertilization rate starting at the lowest tested concentration. This seems to correlate with the reduction of the FC frequency suggesting that TBT affects the mechanism of sperm-oocyte interaction. This evidence is also in agreement with results on other ascidians where oocytes fertilized in presence of TBT resulted in morphological alterations of the extracellular structures that represent the site for sperm recognition, binding and fusion [24,39,41]. Among the mechanisms of action of TBT, it was suggested that an impact on the tubulin, microtubules and conformational changes of structural proteins [14] caused an embryo development arrest. In this study, instead, embryos developed up to larval stage showing an abnormal morphology. In particular, these anomalies concerned the tail that in *C. intestinalis* larvae is a fundamental structure; in fact the tail retraction and modification are crucial processes for the normal metamorphosis from larva to adult [42]. The reduction of FC amplitude induced by TBT may be the cause of abnormal larvae development; in fact, although the functional role of FC has not yet been elucidated, it has been demonstrated that a selective inhibition of FC exerted a long term effect on the embryo development generating either abnormal 8-cell stage embryo known as “rosette” and an abnormal pre-metamorphosis larval formation [26,43].

On the contrary, diuron negatively affects larval development at higher concentrations than TBT that may be attributed to different mechanisms of action.

At last, in this study, according to other authors [23,25,39], toxicity was exerted by using antifoulant concentrations higher than those detected in marine environment (range 1 ng/L–1 µg/L for TBT and 1 ng/L#x2013;6 µg/L for diuron) [20,44,45,46]. However, this may not indicate absence of ecological risk for ascidian populations. In fact, given the worldwide production and extensive uses of antifoulants, the possibility of their accumulation may be also considered.

## 4. Experimental Section

If not otherwise stated, chemicals were purchased from Sigma-Aldrich (Milan, Italy).

### 4.1. Animals and Gametes

Ascidians *C. intestinalis* were collected in the Gulf of Naples (Italy). After collection, animals were maintained in tanks with running seawater at 18 °C. Before use, they were anesthetized in ice and mature oocytes collected with a Pasteur pipette from the oviduct and transferred to Petri dishes containing filtered (Millipore 0.22 µm; Milli Q, Medford, MA, USA) natural seawater (NSW). Spermatozoa were collected with a fine Pasteur pipette from the sperm duct and diluted to 10^6^/mL in NSW before insemination.

### 4.2. Test Solutions

Stock solutions were prepared by dissolving TBT chloride (purity > 96%) and diuron (highest available purity, Residue Analysis-Pestanal) in ethanol and dimethyl sulfoxide (DMSO) respectively.

Test solutions were then obtained by diluting the stock solution in NSW as follows: 1, 2, 3, 4, 5 µM and 10 µM. These concentrations were chosen according to other authors [23,25,39]. As ethanol and DMSO were used to prepare stock solutions, standard control was carried out with equivalent volume at final concentration of 0.1% (that is, the higher one in the test solutions) and exhibited no observable effect on *C. intestinalis* gametes and larval development.

### 4.3. Electrophysiology

Recordings were performed using the whole-cell configuration of the patch-clamp technique at room temperature.

To obtain nude plasma membrane for electrical recordings, the chorion and follicle cells surrounding the oocytes were removed manually using steel needles. Nude oocytes were transferred to the recording chamber containing NSW.

Recordings were performed using a List EPC-7 patch-clamp amplifier (HEKA Electronics, Cologne, Germany), filtered at 3 kHz and digitized with a Digidata 1322A. Currents were acquired and analyzed using pClamp9 software (Axon Instruments, Union City, CA, USA).

Patch pipettes were made from borosilicate glass capillaries (Warner Instruments, Hamden, CT, USA) and pulled using a Sutter P-87 (Sutter Instrument, Novato, CA, USA) with a tip of 1–2 µm in diameter showing a resistance of 3–5 megaOhms when filled with an intracellular-like solution (200 mM K_2_SO_4_; 20 mM NaCl; 200 mM sucrose; 10 mM EGTA; 10 mM HEPES, pH adjusted to 7.5).

Electrophysiological recordings were performed as follows: after formation of a cell-attached patch the application of a light negative pressure induced the rupture of the membrane patch allowing the access to oocyte cytoplasm. In this configuration, from a holding potential of −80 mV, voltage-dependent Na^+^ currents were elicited by applying depolarizing ramps of 10 mV to the test potential from −70 mV to +20 mV, to generate the current-voltage relationship (I/V curves). The FC was generated by adding spermatozoa to the recording bath containing an oocyte voltage clamped at −80 mV.

To evaluate the effect of the biocides on conductance, ion currents and FC the oocytes were incubated for 30 min with test solutions before the electrophysiological recording. For each treatment thirty experiments were performed.

Conductance and the amplitude of ion currents were normalized and reported per mm^2^ of surface area of the oocytes, which was calculated assuming a spherical shape by using the formula 4π*r*^2^.

### 4.4. Toxicity Assays

#### 4.4.1. Exposure of Spermatozoa to TBT and Diuron

Spermatozoa collected from the sperm duct were suspended in NSW at final concentration of 10^6^/mL, aliquots of this suspension were incubated for 30 min at room temperature with test solutions and then used to fertilize untreated oocytes of the same batch.

#### 4.4.2. Exposure of Oocytes to TBT and Diuron

Aliquots of about 200 oocytes, collected from the same animal, were incubated for 30 min at room temperature with the test solutions. Oocytes were then washed twice and transferred into Petri dish containing NSW where they were fertilized with untreated spermatozoa.

#### 4.4.3. Fertilization Test

Aliquots of about 200 oocytes, collected from the same animal, were fertilized with spermatozoa in NSW containing TBT or diuron at concentrations between 1–5 µM and 10 µM and left to develop up to the larval stage in this solution.

#### 4.4.4. Fertilization and Normal Larvae Rate

Fertilization rate was determined, 50 min after fertilization, by assessing the occurrence of the first cleavage (2-cell stage embryos). Then embryos were left to develop up to larval stage in a culture chamber at 18 °C. 24 h after fertilization, morphology of hatched larvae was evaluated at the inverted microscope and the percentage of normal larvae calculated. Each experiment was performed twenty times for both TBT and diuron.

### 4.5. Statistical Analysis

Data were reported as mean ± standard error (SE). In order to test significant differences between the control group and test concentrations and among the test concentration groups we performed one-way analysis of variance (ANOVA) followed by LSD test. In the case of values expressed as percentages, we proceeded to analyze data after arcsine transformation to achieve normality. Significance level was always set at α = 0.05.

## 5. Conclusions

In the present study, we demonstrated that some ascidian reproductive mechanisms are sensitive to antifouling compounds.

TBT has been defined the most toxic substance that have been ever introduced in the environment by human activities [47] and for this reason, an international ban of this compound has led most of the countries to use alternative biocides such as the diuron. Here, we have shown that the latter is less toxic than TBT on some parameters at the base of reproductive processes, however it still exerts a negative effect on the electrical asset of the plasma membrane and on fertilization and larval development. In consideration of the high representative value of *C. intestinalis* [48] among the marine invertebrates, our results substantiate that ascidians may be considered as model organism to test toxicity of pollutants on gametes and embryo [7].
